# Influence of erythropoietin on microvesicles derived from mesenchymal stem cells protecting renal function of chronic kidney disease

**DOI:** 10.1186/s13287-015-0095-0

**Published:** 2015-05-22

**Authors:** Yan Wang, Xingyan Lu, Juan He, Weihong Zhao

**Affiliations:** Department of Geriatrics, the First Affiliated Hospital of Nanjing Medical University, 300 Guangzhou Road, Nanjing, Jiangsu 210029 China

## Abstract

**Introduction:**

Mesenchymal stem cells (MSCs) play a central role in the remediation of cell and tissue damage. Erythropoietin (EPO) may enhance the beneficial influence of MSCs during recovery from tissue and organ injuries. Microvesicles (MVs) released from MSCs contribute to the restoration of kidney damage. We studied the influence of EPO on MVs derived from MSCs, and the protective effects of these factors in subjects with chronic kidney disease (CKD).

**Methods:**

The MVs derived from untreated MSCs (MSC-MVs) or from MSCs incubated in different concentrations of EPO (1, 10, 100, and 500 IU/ml EPO-MVs) were used to treat renal injury of unilateral ureteral obstruction (UUO) in vivo, and transforming growth factor-β1 (TGF-β1)-induced fibrosis in a human renal proximal tubular epithelial (HK2) cell line in vitro. Western blot and reverse transcription polymerase chain reaction (RT-PCR) analyses were used to evaluate the expression of epithelial and mesenchymal markers in the renal tissue and HK2 cells. Flow cytometry was used to assess apoptosis within the HK2 cells, and microRNA (miRNA) microarray assays were used to determine the expression profiles of miRNA in the MSC-MVs and EPO-MVs.

**Results:**

Compared to MSC-MVs (untreated), there was a significant increase in the number of EPO-MVs derived from MSCs treated with 1–100 IU/ml EPO, and these EPO-MVs had a greater benefit in UUO mice on days 7 and 14. Moreover, the EPO-MVs had a better restorative effect following TGF-β1-induced fibrosis in HK2 cells at 24 h and 48 h. The flow cytometry results revealed that both types of MVs, especially EPO-MVs, play an important anti-apoptotic role in HK2 cells treated with TGF-β1. The miRNA profiles of the MVs revealed that EPO-MVs changed 212 miRNAs (fold-change ≥ 1.5), including miR-299, miR-499, miR-302, and miRNA-200, and that 70.28 % of these changes involved upregulation. The changed miRNA in EPO-MVs may have contributed to their enhanced protective effects following renal injury compared to MSC-MVs.

**Conclusions:**

There was a dose-dependent increase in the level of EPO-MVs within the range of 1–100 IU/ml EPO. Although both MSC-MVs and EPO-MVs protect the kidney from fibrosis-related damage, there is a superior effect of EPO-MVs.

**Electronic supplementary material:**

The online version of this article (doi:10.1186/s13287-015-0095-0) contains supplementary material, which is available to authorized users.

## Introduction

Bone marrow-derived mesenchymal stem cells (MSCs), also known as multipotent MSCs, offer a number of potentially exciting effects for the treatment of chronic kidney disease (CKD). Several *in vitro* studies have suggested that MSCs may incorporate into the renal parenchyma, trans-differentiate into new renal tubular cells, and then expand in a straightforward fashion [1–3]. However, in addition to the ability of MSCs to differentiate in the kidney, the beneficial effects of these factors in the cells of injured tissues have also been attributed to their paracrine effects, which indirectly improve renal function via the reduction of disease-related inflammation and fibrosis [4–6]. Accordingly, the paracrine effects of MSCs have recently received more attention for their potential therapeutic effects in CKD patients.

As a one-of-a-kind paracrine factor derived from MSCs, microvesicles (MVs) have been described as a new mode of cell-to-cell communication [7]. MVs interact with target cells via surface-expressed ligands, and act to transfer surface receptors and deliver proteins, messenger RNA (mRNA), microRNA (miRNA), and bioactive lipids. MVs that develop from bone-derived MSCs accelerate recovery following acute kidney injury (AKI) induced by toxic agents [8, 9] or ischemia–reperfusion [10], and induce functional improvements in patients with CKD [11] via miRNA- and mRNA-dependent mechanisms. Because MVs are a paracrine factor, one advantage they have over MSCs is the avoidance of possible long-term maldifferentiation of engrafted cells or tumor generation. In addition, in high concentrations under highly homogeneous preparation conditions, MVs provide focused stimulation that allows for their interaction with target cells and, in turn, positive reparative effects on damaged organs [12].

Erythropoietin (EPO) is a glycoprotein hormone that stimulates the formation and differentiation of erythroid precursor cells in bone marrow via the EPO receptor (EpoR). An increasing amount of evidence has demonstrated that other cell types, including neurons, endothelial cells, cardiomyocytes, and renal tubular cells, also express EpoR and respond to EPO treatment [13]; these findings expand the potential biological roles of EPO beyond erythropoiesis. The majority of in vivo and in vitro experimental studies have shown that EPO protects against acute tissue injury in the brain, heart, and kidney via the activation of relevant signaling pathways that prevent apoptosis and/or stimulate reparative proliferation in the cells of injured tissue [14–17].

Bone marrow-derived cells, both MSCs and endothelial progenitor cells (EPCs), express EpoR and/or mediate the proliferation of cells following EPO treatment [18, 19]. Mice MSCs express EpoRs, and the in vitro application of EPO to these cells prevents apoptotic cell death, whereas its in vivo administration increases the number of MSCs in the bone marrow and spleen [13]. Based on these findings, we investigated the influence of EPO on MVs derived from MSCs (EPO-MVs), and the protective effects of these factors in an in vivo mouse model of CKD. In addition, in vitro experiments were conducted to assess the miRNA expression profile in EPO-MVs that may be responsible for the enhanced effectiveness of this paracrine factor in protecting renal function. This expression was compared to the miRNA expression of MSC-derived MVs (MSC-MVs).

## Methods

### Isolation of mMSC and incubation with EPO

The mice primary bone marrow mesenchymal stem cells (BM-MSCs) were obtained from four-week-old male C57BL/6 mice, cultured and characterized as previously described [20]. The extracted MSCs were cultured to the P3 generation. Then, purified P3-mMSCs were grown under different conditions on six-well culture plates with 10^5^ cells/well: group A (control group), Dulbecco’s modified Eagle’s medium nutrient mixture F-12 (DMEM/F12) with 10 % fetal bovine serum and 2 % penicillin/streptomycin (Hyclone Labs, Logan, UT, USA); group B (different concentrations of the EPO incubation group): Dulbecco’s modified Eagle’s medium nutrient mixture F-12 (DMEM/F12) with 10 % fetal bovine serum and 2 % penicillin/streptomycin (Hyclone Labs) plus different concentrations of EPO (1, 10, 100 and 500 IU/ml for each subgroup, respectively) (Gibco, Frederick, BRL Co. Ltd., MD, USA). Each group was incubated for two days. The extracted cells of group A and group B were identified according to their characteristic of osteogenesis and adipogenesis properties and were also detected using flow cytometry.

### Isolation of MVs and EPO-MVs

The culture medium of each group was collected each time the medium was replaced and then stored at −80 °C to be centrifuged together at 3,000 g for 20 minutes. The free-cell debris supernatants were centrifuged at 100,000 g (Beckman Coulter, Irvine, CA, USA, Optima L-90 K ultra- centrifuge) for 1 h at 4 °C, washed in serum-free medium 199 (M199) containing N-2-hydroxyethylpiperazine-N-2-ethanesulfonic acid (HEPES) 25 mM (Sigma-Aldrich (Shanghai),SHANGHAI, CHINA), ultracentrifuged a second time under the same conditions, and then resuspended with M199. The protein content of MVs from the control group and EPO incubation groups were quantified using the Bradford method (Bio-Rad, Hercules, CA, USA).

### The culture and experimental treatement of HK2

Human renal proximal tubular epithelial cells HK2 (American Type Culture Collection, Manassas, VA, USA) were cultured in Medium 199 powder (Invitrogen, Frederick, MD, USA) supplemented with 10 % fetal bovine serum (Gibco, Burlington, Canada) and 2 % penicillin/ streptomycin (Hyclone Labs). The cells were seeded for 3–4 days at 37 °C in 95 % air–5 % CO2, then trypsinized by 0.25 % trypsin-EDTA (Gibco BRL Co. Ltd.). Cells were growth-arrested in serum-free medium over night before use in experiments. The cells used in this experiment were at passages 10–15. HK2 cells were incubated with recombinant human transforming growth factor-β1 (TGF-β1) (R&D, Minneapolis, MN, USA) (6 ng/ml) for 48 h and 72 h to induce fibrosis with MVs (30 μg) or EPO-MVs (30 μg) treatment.

### Flow cytometry

The Annexin V-FITC apoptosis detection kit (Invitrogen) was used to detect apoptotic cells according to the manufacturer’s protocol. Briefly, cells were gently washed with PBS and collected using trypsinization, disaggregated to a single cell suspension and incubated with 5 μl of Annexin V-FITC and 10 μl of a PI solution for 15 minutes in the dark. The apoptotic cells were detected using flow cytometry (BD, San Diego, CA, USA), then quantified and the percentage of apoptotic cells measured.

### Animal treatments and surgical methods

Animal welfare and experimental procedures were carried out in accordance with the Guide for the Care and Use of Laboratory Animals (Ministry of Science and Technology of China, 2006), and were approved by the animal ethics committee of Nanjing Medical University. Healthy specific pathogen-free (SPF) purpose-bred domestic C57BL/6 mice (6-weeks-old, 15–23 g body weight) were obtained from the Model Animal Research Center of Nanjing University, Jiangsu, China. Animals had ad libitum access to rodent diet and tap water. The mice were kept in cages with a 12:12-h light–dark cycle, a temperature of 21 °C ±2 °C, and a humidity of 55 % ±5 %.

Mice assigned to the unilateral ureteral obstruction (UUO) model were anesthetized by tiletamine/zolazepam (VIRBAC Laboratories, Carros, France) and placed on a heating pad to maintain their temperature at 37 °C. Left ureters were ligated with silk (4/0). Amoxicillin was given to the animals after surgery for three days. Sham animals underwent the same procedure, except that the ureter was not ligated. The animals were randomly divided into four groups: sham group (n = 10), UUO group (n = 10), UUO + MVs group (n = 10), and UUO + EPO-MVs group (n = 10). The mice were injected in the tail vein with MVs or EPO-MVs (30 μg/mouse) one day after surgery The four groups were killed at 7 days and 14 days after UUO.

### Biochemical analysis

Blood and urine were collected before the mice were killed and serum levels of urea nitrogen (BUN) and creatinine (Scr), and urine levels of protein were examined using a Beckman Analyser II (Beckman Instruments, Inc., Fullerton, CA, USA).

### Renal immunostaining and histological analysis

After the mice were killed, the kidneys were fixed in 4 % neutral-buffered paraformaldehyde for histological assessment, embedded in paraffin and cut at 3 μm-thick. Then, the sections were dewaxed using standard sequential techniques before Masson’s trichrome (MT) and hematoxylin-eosin (HE) staining. Damage was assessed as in previous studies [20, 21]. The degree of tubular cell damage was scored by a pathologist, in a single-blind manner, using the numerical Histological Score of Kidney (HSK): 0, no damage; 1, unicellular, patchy isolated necrosis; 2, tubular necrosis less than 25 %; 3, tubular necrosis between 25 % and 50 %; 4, more than 50 % tubular necrosis and presence of infarcted tissue [22].

The sections previously prepared for immunohistochemistry were incubated with primary antibody E-cadherin (1: 800, Bioworld, Louis Park, MN, USA) and α-SMA antibody (1 μg/ml, Abcam, Cambridge, MA, USA) according to the manufacturers’ protocols. After washing, the sections were incubated with secondary antibody, anti-rabbit secondary antibody (Santa Cruz Biotechnology, Dallas, Texas, USA) at a 1:5000 dilution. Finally, sections were counterstained with Mayer’s hematoxylin, dehydrated, and observed.

### Western blotting

Protein was extracted from kidney tissue and cultured cells. The protein concentration was determined using the Bradford method. Then, 40 μg of the extracted protein was electrophoresed on 10 % SDS polyacrylamide gels and transferred to nitrocellulose membranes (Millipore, Bedford, MA, USA). After blocking for 2 h at 4 °C in blocking buffer (10 % fat-free milk TBS with 0.1 %Tween 20), the membrane was incubated overnight with anti-E-cadherin (1:800, Bio-world), anti-α-SMA (1 μg/ml, Abcam), and anti-β-tubulin (1: 5000, Bio-world). The membrane was washed and incubated for 2 h at 4 °C with conjugated anti-rabbit secondary antibody. Detection was performed using enhanced chemiluminescence (Thermo Scientific, Frederick, MD, USA) and photography.

### Reverse transcription and real-time polymerase chain reaction (RT-PCR)

Total RNA was extracted from MVs and HK2 cells according to the Trizol (Invitrogen) manufacturer’s protocol. cDNA was synthesized using the Takara SYBR®PrimeScriptTM miRNA RT-PCR Kit and the PrimeScript RT reagent Kit with gDNA Eraser (Takara, Shiga, Japan). Quantitative real-time PCR was performed using a SYBR PrimeScript miRNA RT-PCR Kit and SYBR Premix Ex TaqII (Tli RNaseH Plus) (Takara) with the intron-spanning primers on ABI-Prism-7500 Sequence Detection System (Applied Biosystems, Frederick, MD, USA). The sequences of the PCR primers of mRNA are: a-smooth muscle actin (α-SMA) –forward -TGTGCTGGACTCTGGAGATG, reverse-ATGTCACGGACAATCTCACG; E-caderin-forward-AGAAGACGCTGAGCATGTGA, reverse-TGGATCCAAGATGGTGATGA; β-actin-forwad-TAAAGACCTCTATGCCAACACAGT, reverse-CACGATGGAGGGGCCGGACTCATC.

### MicroRNA profiling arrays of MV and EPO-MV

To study the differential miRNA expression in MV and EPO-MV, we performed miRNA expression profiling of the culture supernatant of MSC with or without EPO incubation using the miRCURY LNA Array (v.18.0) (Exiqon, Vedbaek, Denmark). RNA samples were labeled with the Exiqon miRCURY Hy3/Hy5 power labeling kit and hybridized on the miRCURY LNA Array (version 18.0) station. Scanning was performed with the Axon GenePix 4000B microarray scanner. GenePix pro version 6.0 was used to read image raw intensity. Replicated miRNAs were averaged and miRNAs at intensities ≥ 30 in all samples were chosen for calculating the normalization factor. Expressed data were normalized using the median normalization. After normalization, differentially expressed miRNAs were identified through fold change filtering. The microarray data were deposited in the NCBI Gene Expression Omnibus (GEO) public repository and are accessible under GEO Series accession number GSE68665.

### Statistical analysis

Results from three independent experiments were expressed as mean ± standard deviation (SD). Comparisons between groups were performed using one-way analysis of variance (ANOVA) in SPSS 17.0. *P* values < 0.05 were considered significant.

## Results

### The characterization of EPO-treated MSCs and MVs

**Fig. 1:**
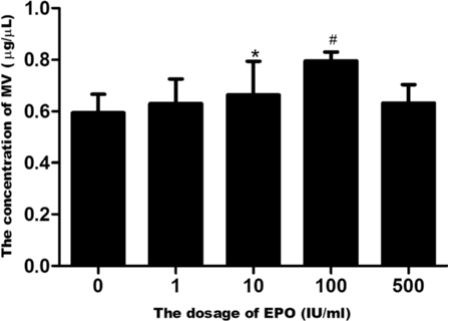
BCA assay changes in the secretion levels of MV derived from MSC after stimulation with different concentrations of EPO for 48 h (n = 3 per group). ^*^ *p* < 0.01 versus 0 IU/ml EPO, ^#^ *p* < 0.01 versus 10 IU/ml EPO. *BCA* bicinchoninic acid *EPO* erythropoietin *MSC* mesenchymal stem cells *MV* microvesicles

**Fig. 2:**
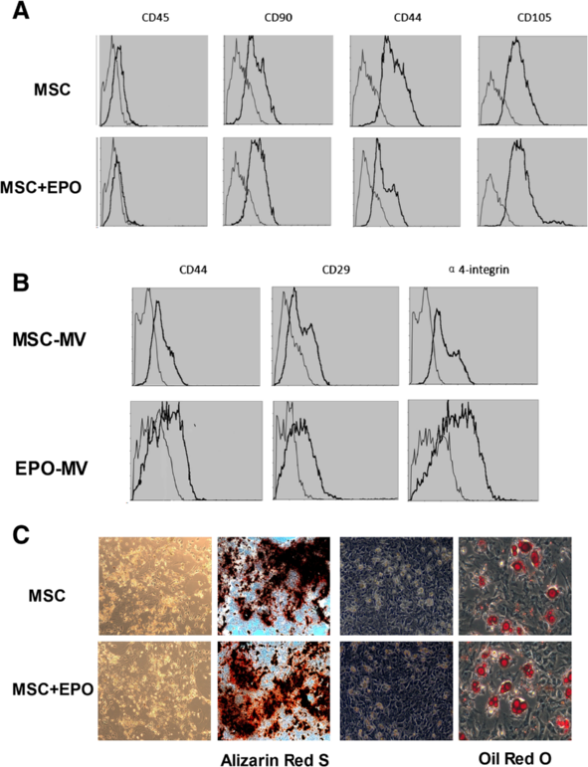
Analysis of the expression of surface markers and characterization of MSCs and EPO-MSC. **a** MSCs and EPO-MSC were labeled with the antibody against CD45, CD105, CD90 and CD44, the cells present were positive for CD105, CD90 and CD44 but negative for CD45. **b** Representative FACS analyses of MSC-MVs and EPO-MVs expressed similar results in CD44, CD29 and α4-integrin. **c** As described in Methods, MSC and EPO incubation MSC(EPO-MSC) undifferentiated, when 50 to 60 or 70 to 80 % confluent, were cultured in conditions that were inducive of osteogenic or adipogenic differentiation, respectively. After osteogenic differentiation, calcium in the mineralized extracellular matrix was shown by Alizarin Red S staining. After adipogenic differentiation, lipid droplets were indicated by their staining with Oil Red O. *EPO* erythropoietin *FACS* fluorescence-activated cell sorting *MSC* mesenchymal stem cells

MSCs were obtained from C57BL/6 mice, and grown in adherent cultures in a plastic culture dish as previously described [20]. All cells were passaged to three generations prior to experimental use, and a subpopulation of secreted MVs induced by the incubation of MSCs in EPO was used; this subpopulation exhibited an optimal concentration of 100 IU EPO/10^6^ MSCs (Fig. 1). Flow cytometry analysis confirmed that the MSCs and EPO-treated MSCs were all CD45-negative and positive in phenotypic markers CD90, CD44, CD105 (Fig. 2a). Cytofluorimetric analyses also showed that both the MSC-MVs and EPO-MVs presented several adhesion molecules known to be expressed on MSC plasma membrane, such as CD44, CD29 and α4-integrin (Fig. 2b).

Marrow stromal cells are often referred to as MSCs due to their ability to differentiate into mesenchymal cell types [23]. This cellular feature distinguishes MSCs from hematopoietic cells and other end-differentiated somatic and progenitor cells that also reside in bone marrow. To verify whether the mesenchymal differentiation capability of the MSCs would change following incubation in EPO, the EPO-treated MSCs were exposed in vitro to specific agents that induce osteogenic or adipogenic differentiation. As shown in Fig. 2c, following exposure to β-glycerol phosphate, ascorbic acid 2-phosphate, and dexamethasone, the undifferentiated cells developed an osteogenic phenotype, whereas after exposure to insulin, dexamethasone, 3-isobutyl-methylxanthine, and indomethacin, the cells manifested an adipogenic phenotype.

### MSC-MVs and EPO-MVs reversed TGF-β1-induced morphological changes in HK2 cells

**Fig. 3:**
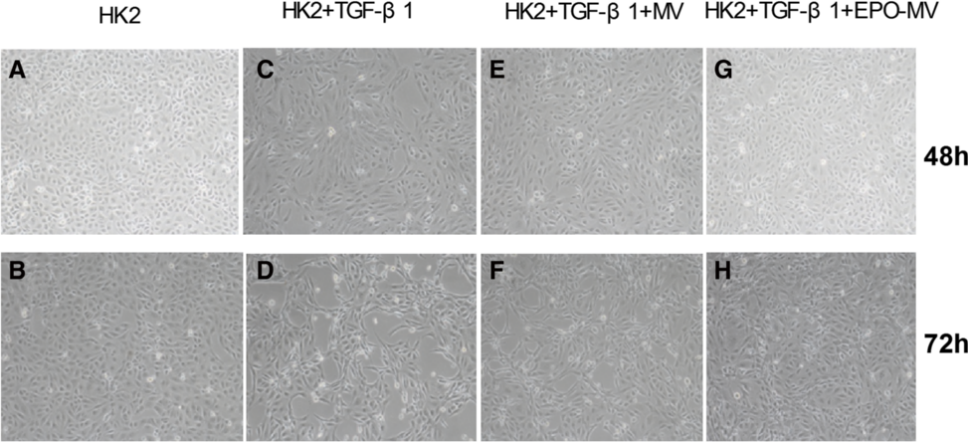
Representative micrographs from each experimental group. **a**-**b** The control group presented polygonal or oval cells, arranged regularly, and showed the characteristics of cobblestone morphology in epithelial cells. **c**-**d** When TGF-β1 (6 ng/ml) was added to these groups to co-culture for 48 or 72 h, the cells lost connection, and the cobblestone morphology was replaced by hypertrophy and long spindle fibroblast-like morphology. Floating cells were increased, and fibrosis injury was more pronounced at 72 h. **e**-**h** MVs/EPO-MVs could partly suppress the HK2 cell morphological changes induced by TGF-β1. Most cells had normal morphology, the degree of fibrosis and its scope were appropriately reduced; in addition, the G-H groups had less fibrosis than the E-F groups. *EPO* erythropoietin *HK2* human kidney 2 *MVs* microvesicles *TGF*-β1 transforming growth factor-β1

To assess the effects of transforming growth factor-β1 (TGF-β1) on cell morphology, HK2 cells were growth-arrested in serum-free medium over night and then treated with TGF-β1 (6 ng/ml) for 48 h or 72 h with or without MSC-MVs or EPO-MVs. Phase-contrast microscopy revealed a decrease in cell-to-cell contacts, a more elongated morphological shape of the cells following TGF-β1 stimulation, and more severe fibrosis at 72 h than at 48 h (Figs. 3c-d). Both types of MVs significantly attenuated the TGF-β1-induced morphological changes, but EPO-MVs had a superior effect (Figs. 3e-h).

### MSC-MVs and EPO-MVs restored the TGF-β1-induced changes in the expression of E-cadherin and α-SMA in HK2 cells

**Fig. 4:**
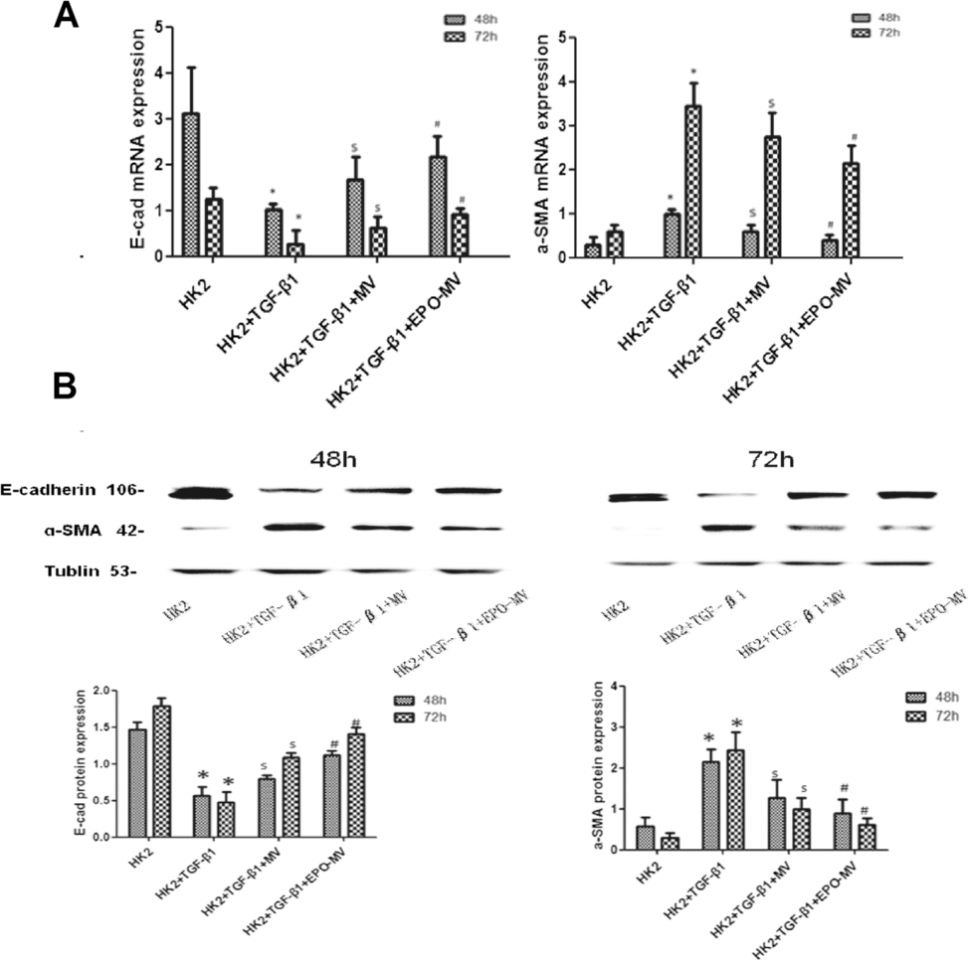
The expression of E-cadherin and α-SMA in HK2 cells. **a** The analysis of gene expression of E-cadherin and a-SMA in HK2 cells following TGF-β1 (6 ng/ml) by RT-PCR at two time points (48 and 72 h) with MVs or EPO-MVs. All of the experiments were repeated three times (n = 3). **b** Western blot was used to detect the protein expression of E-cadherin and α-SMA in HK2 cells following TGF-β1 (6 ng/ml) at two time points (48 and 72 h) with MVs or EPO-MVs. All of the experiments were repeated three times (n = 3).*p < 0.01, 48-h TGF-β1 group versus HK-2 group or 72-h TGF-β1 group versus HK-2 group, ^*$*^ *p* < 0.01, 48-h HK2 + TGF-β1 + MV group versus TGF-β1 group or 72-h HK2 + TGF-β1 + MV group versus TGF-β1 group, ^*#*^ *p* < 0.05, 48-h HK2 + TGF-β1 + EPO-MV group versus HK2 + TGF-β1 + MV group or 72-h HK2 + TGF-β1 + EPO-MV group *versus* HK2 + TGF-β1 + MV. *EPO* erythropoietin *HK2* human kidney 2 *MVs* microvesicles *TGF*-β1 transforming growth factor-β1 *α-SMA* α-smooth muscle actin

To confirm the transformation of the cells into a fibroblast-like phenotype, the expression of E-cadherin and α-smooth muscle actin (α-SMA) in HK2 cells was examined using reverse transcription polymerase chain reaction (RT-PCR) and Western blot analyses. The stimulation of cells with TGF-β1 for 48 h significantly reduced the mRNA levels of E-cadherin in HK2 cells compared to controls; this effect was even larger at 72 h. However, in the presence of MVs, the TGF-β1-induced decrease in E-cadherin mRNA was restored, and the cells exhibited an enhanced reparative ability. In contrast, the expression of α-SMA mRNA exhibited a marked time-dependent increase following stimulation with TGF-β1 at 48 h and 72 h in HK2 cells compared to the control. Both types of MVs reversed this increase, but there was a superior effect with EPO-MVs (Fig. 4a).

The protein levels of E-cadherin and α-SMA, as revealed by Western blot and RT-PCR analyses, showed that both types of MVs restored the TGF-β1-induced changes in E-cadherin and α-SMA in HK2 cells at 48 h and 72 h. However, EPO-MVs exerted a greater reparative influence than MSC-MVs (Fig. 4b).

### MSC-MVs and EPO-MVs inhibited apoptosis in TGF-β1-treated HK2 cells

**Fig. 5:**
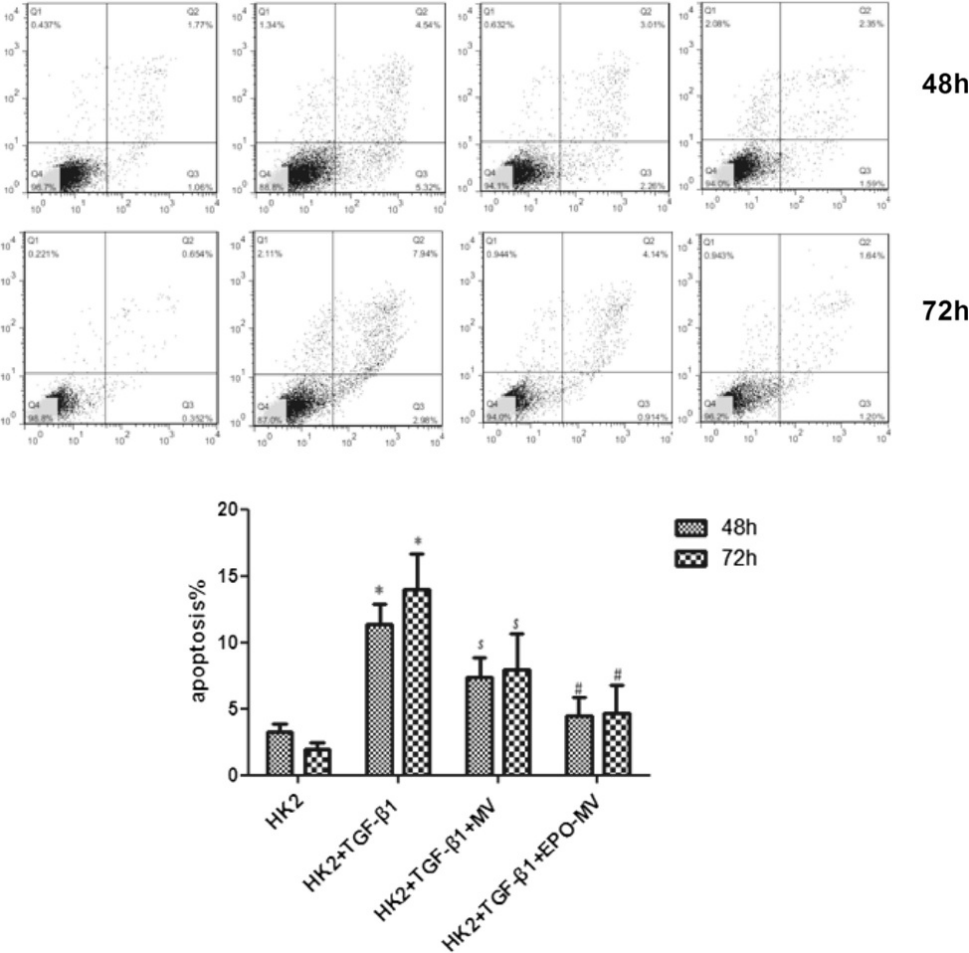
MVs and EPO-MVs inhibited apoptosis of TGF-β treated HK2 cells. Representative photographs of annexinV/PI double-staining in different groups and flow cytometry to test the HK2 cells apoptosis rate after culture in TGF-β1 (6 ng/ml) with MV or EPO-MV at two time points (48 and 72 h) (n = 3 each group). MV and EPO-MV could reduce HK2 cell apoptosis induced by TGF-β1, EPO-MV did better. *p < 0.01, 48-h TGF-β1 group versus HK-2 group or 72-h TGF-β1 group versus HK-2 group, ^*$*^ *p* < 0.01, 48-h HK2 + TGF-β1 + MV group versus TGF-β1 group or 72-h HK2 + TGF-β1 + MV group versus TGF-β1 group, ^*#*^ *p* < 0.05, 48-h HK2 + TGF-β1 + EPO-MV group versus HK2 + TGF-β1 + MV group or 72-h HK2 + TGF-β1 + EPO-MV group versus HK2 + TGF-β1 + MV. *EPO* erythropoietin *HK2* human kidney 2 *MVs* microvesicles *PI* propidium iodide *TGF*-β1 transforming growth factor-β1

To further investigate the anti-apoptotic roles of MSC-MVs and EPO-MVs in HK2 cells following stimulation with TGF-β1, either MSC-MVs or EPO-MVs were co-cultured with TGF-β1 in the HK2 cells for 48 h and 72 h. Flow cytometry analyses revealed that the TGF-β1-induced apoptosis rate of HK2 cells was 9.86 % at 48 h, but the independent application of MSC-MVs and EPO-MVs reduced that rate to 5.27 % and 3.61 %, respectively. At 72 h, the apoptosis rate increased to 10.9 %, but the independent application of MSC-MVs and EPO-MVs reduced that rate to 5.5 % and 2.9 %, respectively. These findings indicate that both types of MVs, but especially EPO-MVs, play an important anti-apoptotic role in HK2 cells treated with TGF-β1 (Fig. 5).

### MSC-MVs and EPO-MVs protected against unilateral ureteral obstruction (UUO)-induced CKD

All subjects in the experimental group survived, and the body weight of each subject was recorded every 3 days until the time of sacrifice. There were no significant differences in body weight observed among the groups (data not shown).

**Fig. 6:**
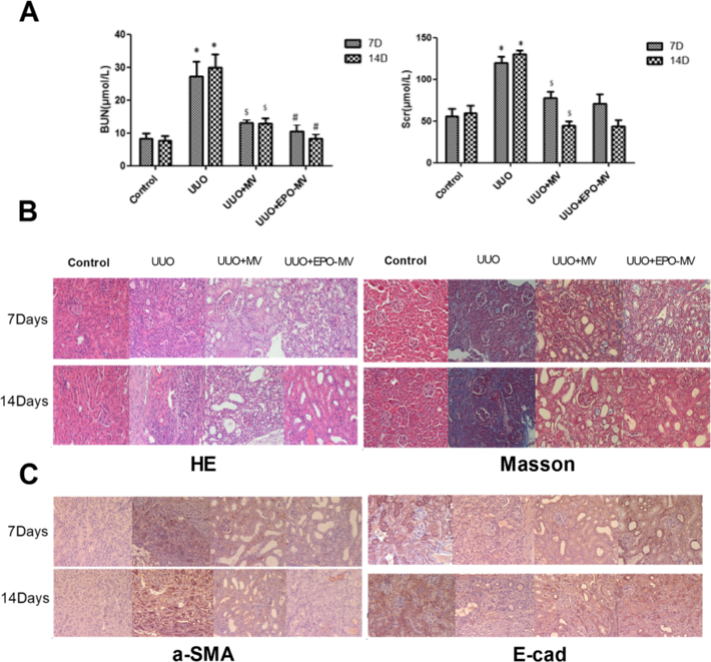
Effect of intravenous injection of MVs or EPO-MVs into unilateral ureteral obstruction (UUO) mice. **a** Mice were subjected to unilateral ureteral obstruction on day 0, followed by intravenous injection of MVs or EPO-MV on day 1 (n = 5 each group). Creatinine and blood urea nitrogen values were determined at the beginning of the experiments and on days 7 and 14 after surgery. *p < 0.01, day 7 UUO group versus control group or day 14 UUO group versus control group, ^*$*^ *p* < 0.01, day 7 UUO + MV group versus UUO group or day 14 UUO + MV group versus UUO group, ^*#*^ *p* < 0.05, day 7 UUO + EPO-MV group versus UUO + MV group or day 14 UUO + EPO-MV group versus UUO + MV group. **b** Representative micrographs of renal histology of the kidney sections with hematoxylin and eosin (HE) and Masson’s trichrome (MT) at days 7 and 14 after surgery in control mice, in UUO mice injected with 30 μg of MVs or EPO-MV. Magnification: ×200. **c** Immunohistochemistry of α-SMA and E-cadherin in renal histology of the kidney sections at days 7 and 14 after surgery in control mice and in UUO mice with or without 30 μg MVs or EPO-MV. *EPO* erythropoietin *MVs* microvesicles *UUO* unilateral ureteral obstruction *α-SMA* α-smooth muscle actin

There were significant increases in serum creatinine and blood urea nitrogen (BUN) levels on days 7 and 14 after the induction of UUO (Fig. 6a). These changes were associated with considerable tubular epithelial injuries that were characterized by tubular dilation, apoptosis, and necrosis, the detachment of tubular epithelial cells, and the presence of proteinaceous casts in the tubules (Fig. 6b). However, there was a significant reduction of the tubular lesions in UUO mice injected with 30 μg MSC-MVs or EPO-MVs 1 day after surgery compared to UUO mice treated with vehicle. The tubular lesions in mice treated with EPO-MVs exhibited markedly better improvement. UUO mice treated with MVs, especially EPO-MVs, also displayed significantly lower levels of serum BUN and urine proteinuria (data not shown) on days 7 and 14 compared to UUO mice treated with vehicle (Fig. 6a).

An assessment of these changes was made using the histological score of kidney (HSK). The kidneys of UUO mice had a score of 2.85 ± 1.0 on day 7 and a score of 3.75 ± 0.5 on day 14, but treatment with both types of MVs restored the morphology of the kidney. Treatment with MSC-MVs resulted in a score of 1.75 ± 0.5 on day 7 and a score of 2.0 ± 0.57 on day 14, whereas treatment with EPO-MVs resulted in a score of 1.17 ± 0.3 on day 7 and a score of 1.16 ± 0.6 on day 14. Each of these scores was statistically significant compared to vehicle-treated UUO subjects (p < 0.05).

Following the UUO procedure, Masson’s trichrome staining revealed a time-dependent increase in extracellular matrix depositions within the tubulointerstitium in which the fibrosis was more severe on day 14. However, both types of MVs resulted in a significant reduction of extracellular matrix deposition in UUO mice, which was greater in the kidneys of mice treated with EPO-MVs (Fig. 6b). An immunohistochemical assessment of α-SMA, a marker of myofibroblasts, demonstrated that there was a time-dependent increase in α-SMA-positive areas in the obstructed kidneys after the UUO procedure. However, the administration of both types of MVs, especially EPO-MVs, resulted in a significant decrease in the α-SMA-positive areas compared to UUO mice on days 7 and 14. On the other hand, E-cadherin, a normal epithelial marker, was markedly reduced in UUO mice. E-cadherin levels were preserved by administration of both types of MVs, especially EPO-MVs (Fig. 6c).

### Expression of α-SMA and E-cadherin in mice kidneys

**Fig. 7:**
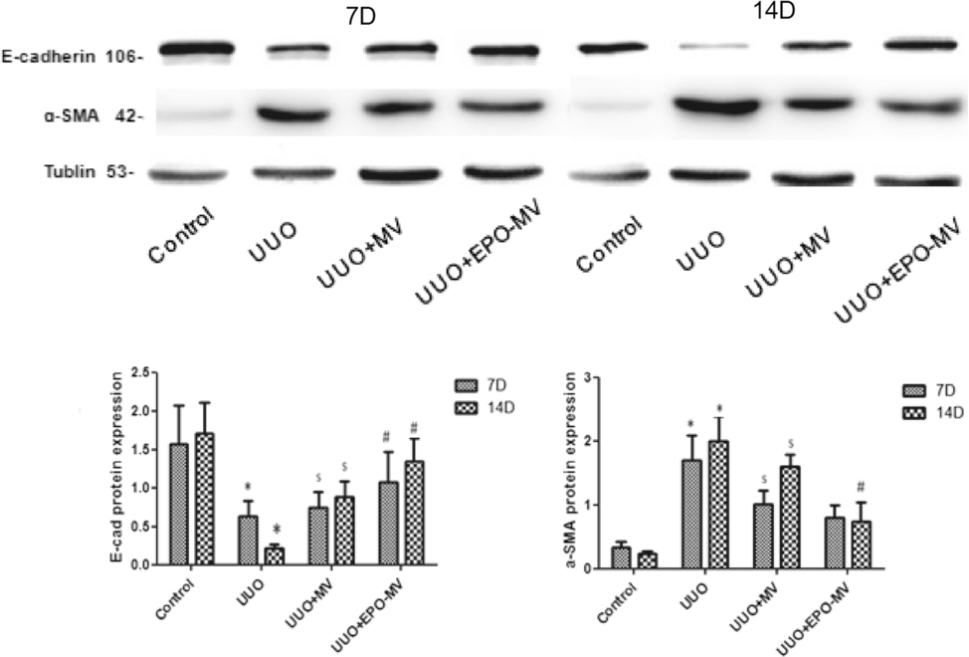
The expression of E-cadherin and α-SMA following UUO and MVs/EPO-MVs administration. Western blot analysis was used to detect the protein expression of E-cadherin and a-SMA as a percentage of tubulin in mice exposed to control and MV/EPO-MV administration at 7 and 14 days. All of the experiments were repeated three times (n = 3). *p < 0.01, 7 day UUO group versus control group or 14 day UUO group versus control group, ^*$*^ *p* < 0.01, 7 day UUO + MV group versus UUO group or 14 day UUO + MV group versus UUO group, ^*#*^ *p* < 0.05, 7 day UUO + EPO-MV group versus UUO + MV group or 14 day UUO + EPO-MV group versus UUO + MV group. *EPO* erythropoietin *MVs* microvesicles *UUO* unilateral ureteral obstruction *α-SMA* α-smooth muscle actin

The expression levels of α-SMA and E-cadherin were determined by Western blot analyses. The expression of α-SMA was significantly higher in the kidneys of UUO mice compared to control mice on days 7 and 14, and EPO-MVs were more effective than MSC-MVs in restoring these levels. On the other hand, the expression of E-cadherin was lower in UUO mice on days 7 and 14, but EPO-MVs were also more effective than MSC-MVs in restoring these levels (Fig. 7).

### Changes in the miRNA profiles of MSC-MVs and EPO-MVs

**Fig. 8:**
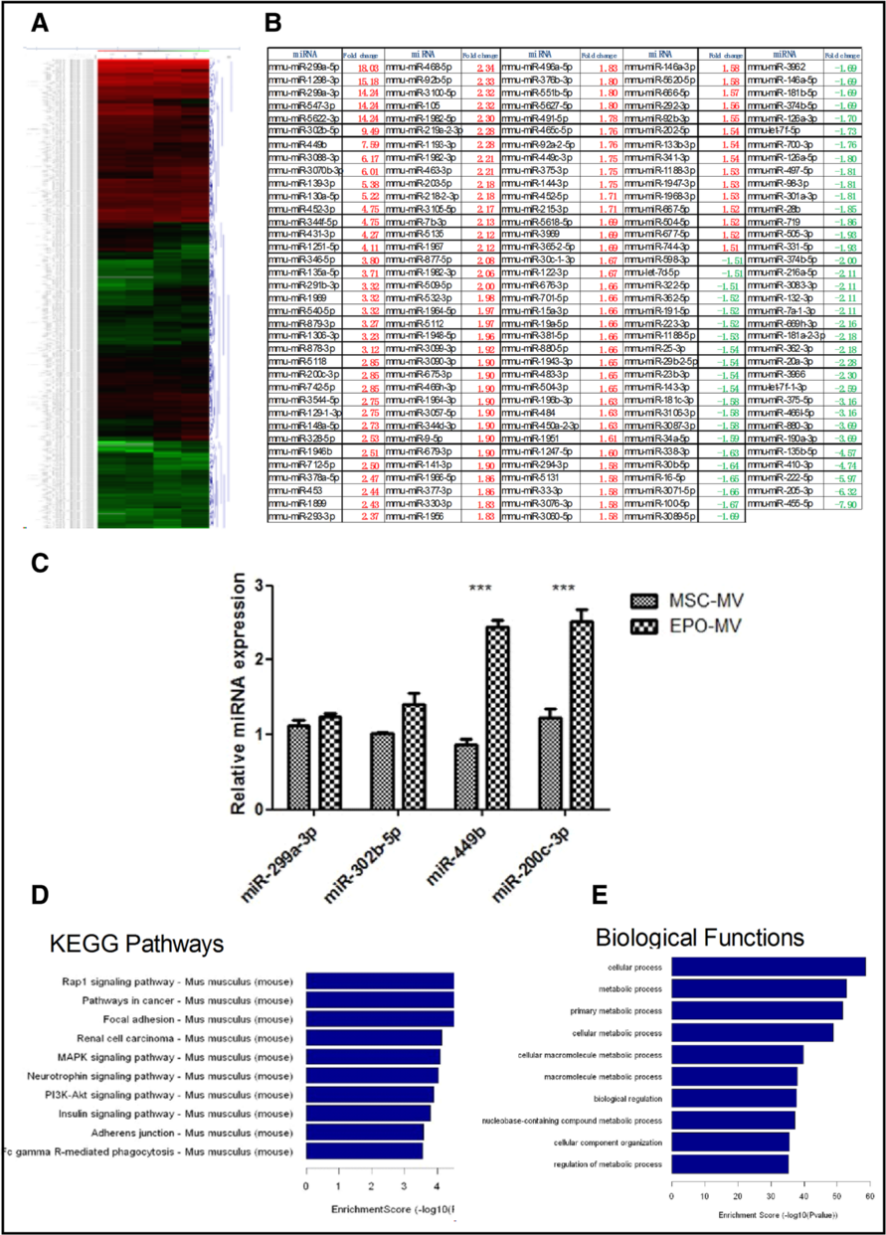
Changes in miRNA profiles of microvesicles (MVs) and EPO treated microvesicles (EPO-MVs). **a** Expression of miRNA in MVs and EPO-MVs, and mapping of hierarchical clustering of miRNA and heat map display of miRNA profiles. Columns represent individual samples, and each row represents individual assayed miRNA. Green represents down-regulation of miRNAs, and red represents up-regulation of miRNAs. **b** Fold regulation of significant miRNA in MVs versus EPO-MVs. Up-regulated miRNAs are denoted in red, downregulated miRNAs are green. **c** The relative miRNA change in MVs and EPO-MVs. The miR-299a-3p, miR-302b-5p, miR-499 and miR-200c-3p were all up-regulated in EPO-MVs; the up-regulation of miR-499 and miR-200c-3p was significant (*p* < 0.001, n = 3). **d** The bar plot shows the top ten enrichment score value of the significant enrichment pathway of the predicted possible target genes of miR-299a-3p, miR-302b-5p, miR-499 and miR-200c-3p. **e** The plot shows top ten biologic functions from the predicted possible target genes of miR-299a-3p, miR-302b-5p, miR-499 and miR-200c-3p, P-value cutoff (*p* < 0.05). *EPO* erythropoietin *miRNA* microRNA

Scanned images of the MSC-MVs and EPO-MVs were imported into GenePix Pro 6.0 software (Axon) for grid alignment and data extraction. The replicated miRNAs were averaged, and miRNAs with intensities ≥ 30 in all samples were used to calculate the normalization factor. The expressed data were normalized with median normalization, and the differentially expressed miRNAs were identified using fold change filtering. Subsequently, the miRNA profiles of the MSC-MVs and EPO-MVs were analyzed with a quantitative PCR (qPCR)-based array of the whole mice genome. Further analysis revealed differences in the miRNAs of 212 EPO-MVs (fold change ≥ 1.5 compared to the MSC-MVs), which constituted approximately 22.64 % of all the evaluated mouse miRNAs. Of all the differences, 70.28 % of the changes in the EPO-MV group involved upregulation (Fig. 8b); this was greater than the changes in the MSC-MV group.

## Discussion

In recent years, MSCs have received an increasing amount of attention in the field of regenerative medicine because they possess a number of potential therapeutic applications for patients with acute and chronic tissue injury in organs such as the heart, kidneys, lungs and liver. In fact, MSCs are now used in clinical trials to treat a wide range of diseases [24]. Although MSCs have multiple differentiation potentialities and migrate to injured sites following systemic administration, several recent studies have indicated that the differentiation of MSCs in the cells of injured tissues has little therapeutic benefit [5, 25, 26]. In fact, a large amount of evidence indicates that the therapeutic effects of MSCs are dependent on their paracrine functions, which allow for the secretion of soluble factors that repair damaged tissues [27–29]. For example, MVs released from MSCs are known to contribute to tissue repair in various animal models of injury [11, 30, 31].

MVs are defined as a vesicle population that is a mixture of exosomes and shedding vesicles that can be detected both in vitro and in vivo. These factors are instrumental in cell-to-cell communication, the interaction with cells via specific receptor–ligand interactions, and the transfer of surface receptors, proteins, and bioactive lipids to target cells after internalization [12]. In addition, MVs contain selected mRNA and miRNA profiles, and aid in the exchange of genetic information between cells [32–34]. MSCs release a significant amount of MVs with mRNA and selected patterns of mature miRNAs that protect against acute and chronic kidney disease [10, 11, 20]. In contrast to MSCs, MVs transfer complex and biologically active substances derived from MSCs to injured cells that favor tissue regeneration [7]. The repeated administration of allogeneic MVs that are derived from MSCs may not involve the immune response, as they do not express histocompatibility antigens.

Along with stem cell-based therapy, EPO has recently been evaluated for the treatment of tissue injury due to its novel pharmacological effects, because in addition to its recognized erythropoietic function, EPO may protect tissues against various types of damage [35]. EPO enhances regeneration of the tubular epithelium via its anti-apoptotic and anti-inflammatory features [36], prevents acute kidney injury (AKI), and improves postoperative renal function when administered in a preventive fashion [37]. Several studies have reported that EPO enhances the ability of MSCs to treat renal and heart injuries [38, 39]. Based on such findings, the present study investigated the influence of MVs derived from EPO-treated MSCs, and evaluated the renal protective function of this type of MV following chronic injury both in vivo and in vitro.

Recent studies have shown that EPO regulates the formation of red blood cells and the proliferation of EPCs [40], gastric epithelial cells [41], and other non-hematopoietic cells that express EpoRs. Using the Bicinchoninic acid (BCA) assay and Cell Counting Kit-8 (CCK-8), we found that the level of MVs secreted from MSCs increased in a significant and dose-dependent manner when treated with EPO concentrations between 1–100 IU/ml (Fig. 1). However, light microscopy data indicated that 500 IU/ml EPO resulted in the obvious death of MSCs, and that there was a marked decrease in the optical density (OD) of the group receiving this dose of EPO (see Additional file 1). Based on these findings, it appears that the significant and dose-dependent EPO-induced increase in MVs can be attributed to the proliferation of MSCs.

Our findings demonstrate that, although both types of MVs had a beneficial effect, the influence of EPO-MVs was considerably stronger than those of MSC-MVs. According to the blood and urine examinations of UUO mice that received MVs 1 day after surgery, the serum levels of BUN in UUO mice exhibited a marked decrease following both MSC-MV (*p* < 0.01) and EPO-MV (*p* < 0.01) administration, but there was a stronger effect of EPO-MVs on days 7 and 14 (*p* < 0.05). In addition, both types of MVs significantly reduced the serum creatinine levels of UUO mice compared to vehicle-treated UUO mice (*p* < 0.01), but the difference in serum creatinine levels between the EPO-MV and MSC-MV groups was not statistically significant. Similarly, both types of MVs markedly reduced the elevated levels of proteinuria in UUO mice on days 7 and 14, but EPO-MVs had a stronger effect (data not shown).

The kidney sections of UUO mice treated with MVs that were stained with hematoxylin and eosin or Masson’s trichrome demonstrated a relatively preserved cytoarchitecture, and a reduction of the histological features of renal injury. However, the mice treated with EPO-MVs displayed fewer interstitial lymphocyte infiltrates, less tubular swelling, less necrosis, and lower levels of interstitial collagen deposition over time. These findings indicate that a higher number of E-cadherin-positive and a lower number of α-SMA-positive cells were detected in the renal tubules and capillaries of mice treated with EPO-MVs compared to mice treated with MSC-MVs. The in vitro experiments further supported the beneficial effects of treatment with MSC-MVs and EPO-MVs. Both types of MVs reversed the TGF-β1-induced epithelial–mesenchymal transition (EMT) in HK2 cells and inhibited the apoptosis of TGF-β1-treated HK2 cells. Once again, EPO-MVs had a stronger effect than MSC-MVs.

A previous study from our group found that MVs ameliorated the renal damage observed in five of six nephrectomized mice and protected kidneys in the same manner as MSCs [20]. These protective effects are likely due to the MV-controlled shuttling of particular miRNAs during renal cell regeneration. This shuttling of factors has been observed both in vivo and in vitro in association with MVs derived from EPCs that have been subjected to a knockdown of Dicer, an intracellular enzyme essential for the production of miRNA [10]. In addition, clinical and experimental studies have provided data that support a critical role for miRNAs in renal pathophysiology. In a mouse model of diabetic nephropathy, inhibition of miR-21 improved kidney function and alleviated the progression of renal injury [42], whereas inhibition of renal miR-192 suppressed the expression of fibrotic markers, such as collagen, TGF-β1, and fibronectin in a mouse model of type I diabetes nephropathy [43].

To explain the improved reparative effects associated with MVs observed in the present study, a miRCURY™ LNA Array was conducted to assess whether the incubation of cells in EPO changes the expression of miRNAs in MVs. The present findings demonstrate that greater numbers of miRNAs (70.28 %) were upregulated in EPO-MVs. Several recent reports have described miRNA functions and their role in anti-fibrosis. miR-302 decreases TGF-β1-induced EMT in renal HKC8 epithelial cells and attenuates the TGF-β1-induced mesangial production of fibronectin and thrombospondin [44]. In vitro studies have demonstrated that members of the miRNA-200 family inhibit the E-cadherin transcriptional repressors ZEB1 and ZEB2, which are implicated in EMT [45]. miR-299 may delay or protect against replicative senescence via improvements in the metabolic activity of senesced cells, but it does not stimulate growth in the remaining senescent cells [46]. miR-499 plays an inhibitory role in the mitochondrial apoptosis pathway, and protects against H_2_O_2_-induced injury in cardiomyocytes [47]. Our findings also demonstrate that miR-299, miR-499, miR-302, and miRNA-200 were upregulated in EPO-MVs (Fig. 8c). Thus, it is likely that the changes in these miRNAs in EPO-MVs contributed to the enhanced inhibition of TGF-β1-induced EMT, decreased levels of apoptosis in the proximal tubular epithelial cells, and improved renal function in UUO mice.

There were several limitations in the present study. Therefore, further investigation regarding the role of upregulated miRNAs in EPO-MVs is required to gain a better understanding of the mechanisms underlying MSC-induced renal protection. Nevertheless, these findings will help guide future research in this area.

### Limitation

The MSCs were cultured in normal 10 % FBS, and then the MSC-MVs and EPO-MVs were collected. The process of isolating and collecting MSC-MVs and EPO-MVs was the same, and, therefore, the influence of FBS on MSC-MVs and EPO-MVs was not only similar, but mitigated to some extent, which allowed for a comparison of their reparative effects on CKD.

## Conclusions

Our findings suggest that there was a dose-dependent increase in the level of EPO-MVs within the range of 1–100 IU/ml EPO, the EPO-MVs had a greater benefit in UUO mice and had a better restorative effect following TGF-β1-induced fibrosis in HK2 cells. The changed miRNA in EPO-MVs may have contributed to their enhanced protective effects following renal injury compared to MSC-MVs. Our findings may help to develop new therapeutic MVs to CKD caused by renal fibrosis in further work.

## Additional files

Additional file 1:
**The OD value in EPO-MSC.**

## Abbreviations

AKI: acute kidney injury
ANOVA: one-way analysis of variance
BCA: bicinchoninic acid
CKD: chronic kidney disease
DMEM/F12: Dulbecco’s modified Eagle’s medium nutrient mixture F-12
EPCs: endothelial progenitor cells
EPO: erythropoietin
EPO-MVs: microvesicles derived from MSCs incubated with EPO
EpoR: EPO receptor
HE: hematoxylin-eosin
HK2: human renal proximal tubular epithelial cell
HSK: Histological Score of Kidney
miRNA: microRNA
MSC-MVs: microvesicles derived from MSCs
MSCs: mesenchymal stem cells
MT: Masson’s trichrome
MVs: microvesicles
RT-PCR: reverse transcription polymerase chain reaction
TGF-β1: transforming growth factor-β1
UUO: unilateral ureteral obstruction
α-SMA: α- smooth muscle actin
