# Supplementary figures and images for: Influence of erythropoietin on microvesicles derived from mesenchymal stem cells protecting renal function of chronic kidney disease
Source: Stem Cell Res Ther. 2015 May 22;6(1):100. doi: 10.1186/s13287-015-0095-0 (PMC4469245; doi:10.1186/s13287-015-0095-0)

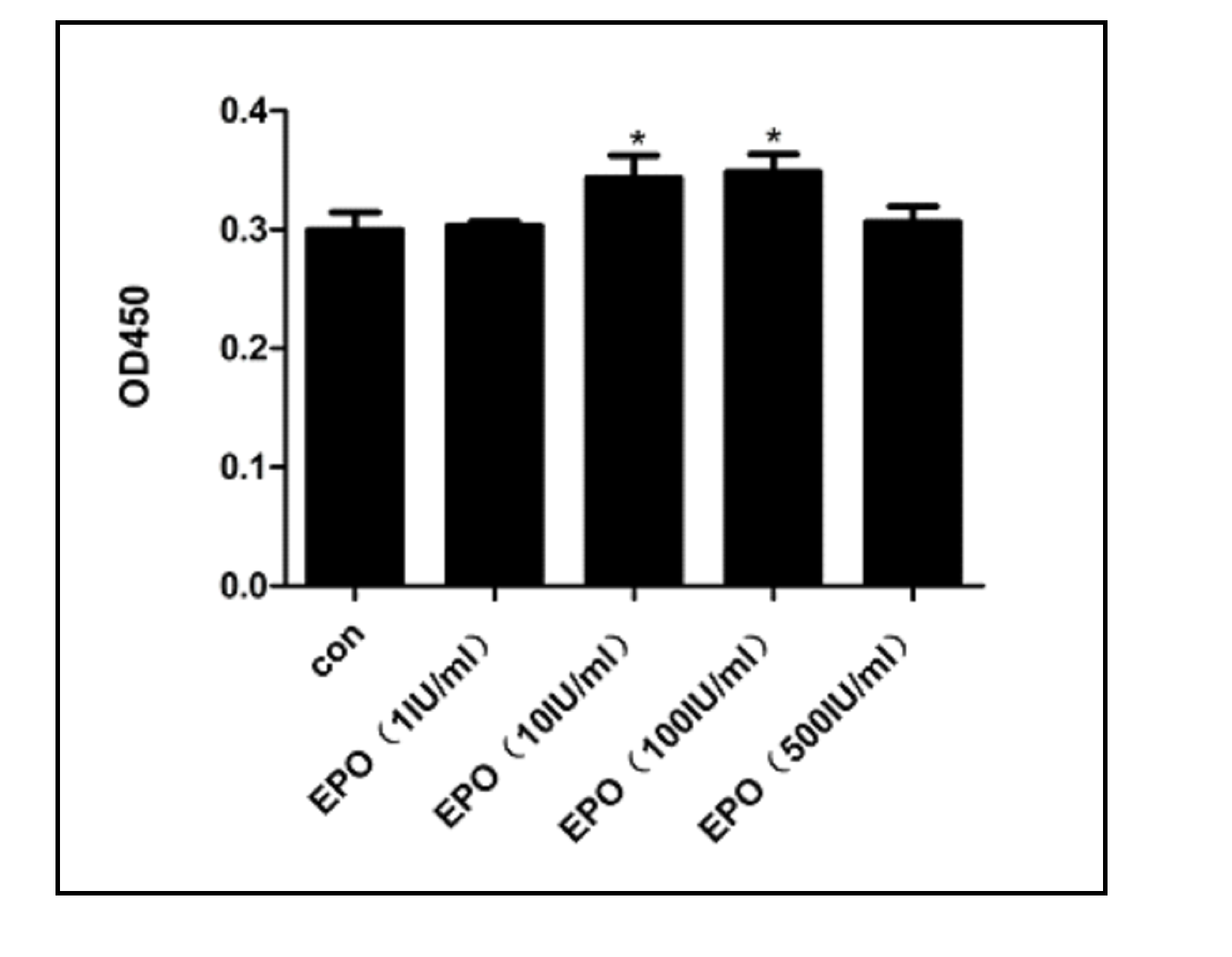

Supplement: Additional file 1: — The OD value in EPO-MSC. [file 13287_2015_95_MOESM1_ESM.tiff]
